# A GWAS assessment of the contribution of genomic imprinting to the variation of body mass index in mice

**DOI:** 10.1186/s12864-015-1721-z

**Published:** 2015-08-05

**Authors:** Yaodong Hu, Guilherme JM Rosa, Daniel Gianola

**Affiliations:** Department of Animal Sciences, University of Wisconsin - Madison, 1675 Observatory Dr., Madison, 53706 WI USA; Department of Biostatistics and Medical Informatics, University of Wisconsin - Madison, 600 Highland Avenue, Madison, 53792 WI USA; Department of Dairy Science, University of Wisconsin - Madison, 1675 Observatory Dr., Madison, 53706 WI USA

**Keywords:** Epigenetics, Genomic imprinting, Genome-wide association studies, Missing heritability, Mouse body mass index

## Abstract

**Background:**

Genomic imprinting is an epigenetic mechanism that can lead to differential gene expression depending on the parent-of-origin of a received allele. While most studies on imprinting address its underlying molecular mechanisms or attempt at discovering genomic regions that might be subject to imprinting, few have focused on the amount of phenotypic variation contributed by such epigenetic process. In this report, we give a brief review of a one-locus imprinting model in a quantitative genetics framework, and provide a decomposition of the genetic variance according to this model. Analytical deductions from the proposed imprinting model indicated a non-negligible contribution of imprinting to genetic variation of complex traits. Also, we performed a whole-genome scan analysis on mouse body mass index (BMI) aiming at revealing potential consequences when existing imprinting effects are ignored in genetic analysis.

**Results:**

10,021 SNP markers were used to perform a whole-genome single marker regression on mouse BMI using an additive and an imprinting model. Markers significant for imprinting indicated that BMI is subject to imprinting. Marked variance changed from 1.218 ×10^−4^ to 1.842 ×10^−4^ when imprinting was considered in the analysis, implying that one third of marked variance would be lost if existing imprinting effects were not accounted for. When both marker and pedigree information were used, estimated heritability increased from 0.176 to 0.195 when imprinting was considered.

**Conclusions:**

When a complex trait is subject to imprinting, using an additive model that ignores this phenomenon may result in an underestimate of additive variability, potentially leading to wrong inferences about the underlying genetic architecture of that trait. This could be a possible factor explaining part of the missing heritability commonly observed in genome-wide association studies (GWAS).

## Background

Genomic imprinting, an epigenetic process, is the preferential or differential gene expression in a parent-of-origin fashion [1, 2]. If the expression of the maternally (or paternally) inherited allele is “switched off” (i.e., the allele is silenced), it is called maternal (or paternal) imprinting and this complete silence represents the canonical definition of imprinting. In cases where gene expression is not completely repressed, the phenomenon is called partial, as opposed to complete, imprinting [3, 4]. The regulation of genomic imprinting is not fully understood yet, but the mechanism is usually thought to be caused by differential epigenetic modification (mainly DNA methylation and histone modification) of two parental genomes [5]. The emergence and evolution of genomic imprinting is a puzzle to geneticists since the functional haploidy caused by imprinting may increase the risk of being exposed to a deleterious mutation, unlike the case of Mendelian inheritance where there is always a “backup” compensation from the other allele. Although many hypotheses have been offered (e.g., all articles in the journal Heredity, vol. 113, issue 2, Aug. 2014 were on evolutionary theories of imprinting), only the parent-offspring conflict hypothesis that relates imprinting to nutrient resources allocation of alleles with different parent-of-origin has been widely accepted [6, 7]. Despite of pending questions on the mechanisms and evolution of imprinting, it is widely believed that imprinting can affect many complex traits [8], including economically important traits in agricultural animal and plant species [9–12] as well as human diseases like the Prader-Willi (PWS) and Angelman (AS) syndromes [13, 14]. Thus, relating phenotypes of complex traits to (epi)genetic variants is of interest.

Studies of complex traits often aim at partitioning phenotypic variance into different components. In classical quantitative genetics (e.g., [15]), the phenotypic variance is partitioned into the sum of genotypic and environmental variances, and the genotypic variance is further subdivided into additive and dominance variances. If two or more loci are involved, there could also be an epistatic variance component. The ratio between the additive genetic and the phenotypic variances (${\sigma ^{2}_{A}}$ and ${\sigma ^{2}_{P}}$, respectively) defines the narrow sense heritability (*h*^2^). Therefore, *h*^2^ is usually interpreted as the proportion of phenotypic variance explained by additive variance. This parameter is also the expected fraction of the selection differential transmitted from the parental to the offspring generation, and is crucial in artificial selection [15]. Hence, knowledge of *h*^2^ is important in genetic improvement programs for predicting breeding values of selection candidates, and it has been conventionally estimated using kinship information under a linear mixed effect model specification [16].

In recent years, the advent of SNP (single nucleotide polymorphisms) markers made it possible to perform genetic analysis at the DNA level as well as to carry out genome-wide association studies (GWAS), with the goal being finding genomic regions that potentially have an effect on a complex trait of interest. In GWAS, additive variation has attracted most attention while dominance has been largely ignored, as it is deemed not to contribute to heritable variation under a classical quantitative genetics framework (e.g., [15, 17]). However, unlike dominance or epistasis involving dominance, an imprinting effect is thought to be transmissible over generations [2, 18]. This suggests that imprinting may contribute to the additive genetic variance and that ignoring existing imprinting may lead to erroneous inference of genetic variation. In what follows, therefore, we first make a brief review of a one-locus genetic model with consideration of imprinting. This model was proposed in three different studies published in the same year [18–20]. We then discuss the contribution of genomic imprinting to genetic variation by means of a stylized analysis based on this imprinting model. A GWAS-like analysis on body mass index (BMI) in a sample from a mouse population was conducted to assess potential consequences if existing imprinting is ignored in genetic analysis. This paper ends with a discussion on our findings and related issues.

### A one-locus imprinting model

In a standard quantitative genetic model without imprinting, three genotypic values −*a*, *d* and *a* can be assigned to three possible genotypes *A*_2_*A*_2_, *A*_1_*A*_2_/*A*_2_*A*_1_ and *A*_1_*A*_1_ respectively, in a biallelic locus. With imprinting, on the other hand, the resulting parent-of-origin effect makes the two reciprocal heterozygotes different from each other. Hence, the four genotypes *A*_2_*A*_2_, *A*_1_*A*_2_, *A*_2_*A*_1_ and *A*_1_*A*_1_ should be all uniquely identifiable and four different genotypic values are needed in this case. This constitutes the basic configuration of a model incorporating imprinting.

**Fig. 1:**
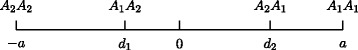
Genotypic values of the four possible genotypes in a biallelic locus with imprinting [18]

Three studies have proposed this model concurrently [18–20], with the only difference being in parameterization. In [18], genotypic values −*a*, *d*_1_, *d*_2_ and *a* are assigned to the four genotypes as shown in Fig. 1 (maternally inherited allele written first). If *p* and *q* are the allele frequencies of *A*_1_ and *A*_2_ alleles, respectively, these four genotypes will have frequencies *q*^2^, *pq*, *qp* and *p*^2^ if Hardy-Weinberg equilibrium holds, and basic statistics gives the mean and variance of the genetic values as *μ*_*G*_=*a*(*q*−*p*)+2*pqd* and ${\sigma ^{2}_{G}} = 2pq(a+d(q-p))^{2} + (2pqd)^{2} + 2pqi^{2}$. Shete and Amos [19] adopted the same parameterization as [18], but also set *d*=(*d*_1_+*d*_2_)/2 as dominance effect in the conventional sense and *i*=(*d*_2_−*d*_1_)/2 as the imprinting effect, so that *d*_1_=*d*−*i* and *d*_2_=*d*+*i*, which was also suggested by [20]. These two parameterizations are equivalent since they are linearly related. However, the parameterization *a*, *d* and *i* of [19] keeps the conventional dominance effect parameter *d* and defines a new parameter *i* as imprinting effect, which simplifies understanding because the imprinting effect is defined explicitly. Thus, we adopt this parameterization.

### Paternal and maternal effects of allele substitution

When *A*_1_*A*_2_ and *A*_2_*A*_1_ are not treated distinctly, *α*=*α*_1_−*α*_2_=*a*+(*q*−*p*)*d* is the average effect of gene substitution with *α*_1_ and *α*_2_ being the allelic effects of *A*_1_ and *A*_2_ alleles, respectively; here *p*= Pr(*A*_1_) and *q*=1−*p*. This value can be derived either by calculating the difference between the genotypic mean of individuals with at least one *A*_1_ (*A*_2_) allele and the population mean in a randomly mating population, as shown in [15], or via a linear regression approach as described in [17].

When imprinting is considered, however, two such substitution effects are needed since the same allele could have a different effect when it is inherited from the father or the mother, due to the fact that the expression of one allele is repressed in case of imprinting. Spencer [18] adopted the approach in [15] and analytically deduced the eight breeding values (four genotypes with each acting as a sire or a dam) in an idealized population. Using the relationship between breeding value and substitution effect shown in [15], two parental substitution effects were obtained. Shete and Amos [19], on the other hand, followed the approach in [17] with the linear model
(1)

where *G*_*j*_={−*a*,*d*−*i*,*d*+*i*,*a*} is the genotypic value of each of the four genotypes; *I*_*♂*_ and *I*_*♀*_ are (0,1) indicator variables denoting the number of *A*_1_ alleles inherited from a specific parent (e.g., *I*_*♂*_=0 and *I*_*♀*_=1 denotes that the genotype is *A*_1_*A*_2_ with the maternally inherited allele written first); *δ*_*j*_ is the model residual, interpreted as dominance deviation in [17]. In Model 1, *α*_*♂*_ and *α*_*♀*_ are the substitution effects in two parental lines. Both [18, 19] arrived at *α*_*♂*_=*a*+*i*+(*q*−*p*)*d* and *α*_*♀*_=*a*−*i*+(*q*−*p*)*d* as paternal and maternal allele substitution effects, respectively. Note that (*α*_*♂*_−*α*_*♀*_)/2=*i*, the imprinting effect, and (*α*_*♂*_+*α*_*♀*_)/2=*α*, the average gene substitution effect in the standard sense. de Koning *et al.* [20] presented the same result.

## Contribution of imprinting to genetic variation

The potential role of epigenetics on complex traits has led to studies of the impact of epigenetic variation on phenotypic and genetic variations. For example, in a recent *in silico* study, it was shown that epigenetic modification of one allele at a biallelic locus can result in an 11 % of total genetic variance attributed to epigenetic variation at moderate allele frequency even if *u*, the epigenetic modification rate, is as low as 0.01 [21]. The proportion of genetic variance explained by epigenetic variation could be as large as 18 % if *u* increases to 0.5. This can be explained by viewing the epigenetic modification as producing an epi-mutation that has a similar effect as a regular mutation event if the epi-mutation persists a relatively long time in a population, i.e., if transmissible between generations.

Transmissible variation is very important in breeding programs, since it determines the mean performance of the offspring generation after applying artificial selection to the parental generation. In a quantitative genetics context, transmissible variation consists of the additive genetic variance ${\sigma ^{2}_{A}}$, which defines narrow sense heritability *h*^2^ through the ratio ${\sigma ^{2}_{A}}/{\sigma ^{2}_{P}}$ as stated above, where ${\sigma ^{2}_{P}}$ is the phenotypic variance. Additive genetic variance has been traditionally estimated using phenotypic records and pedigree information with likelihood-based or Bayesian methods [16, 22–26], but in the genomic era, one can usually estimate the substitution effect of an allele at some known locus and use an estimate of 2*pqα*^2^ as the additive variance contributed by that locus, if Hardy-Weinberg equilibrium holds.

If imprinting is involved in the analysis, the two parental substitution effects *α*_*♂*_ and *α*_*♀*_ can be used to calculate the additive genetic variance in a similar way as in the additive model. Here, the paternal and maternal contributions to the additive variation can be separated due to different substitution effects of a paternally inherited and maternally inherited allele. According to [18, 19], the additive variance under imprinting is given by
(2)

if a 1:1 sex ratio is assumed. Note that the first term in Eq. 2 is the additive variance under an additive model without imprinting; and when imprinting is considered, an extra term 2*pqi*^2^ is added to ${\sigma ^{2}_{A}}$. Same as in an additive case, ${\sigma ^{2}_{A}}$ under imprinting can be derived by taking the variance of breeding values of all eight genotypes (4 possible genotypes and 2 sexes) [18], and hence both 2*pq*(*a*+(*q*−*p*)*d*)^2^ and 2*pqi*^2^ represent the transmissible variance over generations. Therefore, for the sake of clarify, we will call 2*pq*(*a*+(*q*−*p*)*d*)^2^+2*pqi*^2^ as the additive genetic variance, because this is the variance between breeding values under imprinting. Parts 2*pq*(*a*+(*q*−*p*)*d*)^2^ and 2*pqi*^2^ are referred to as Mendelian (i.e., the unimprinted part) and imprinting variances, and are denoted by $\sigma ^{2}_{\textit {Men}}$ and $\sigma ^{2}_{\textit {Imp}}$, respectively [27]. Since *i* enters into the additive genetic variance, imprinting contributes to narrow sense heritability, as well as to total genetic variance ${\sigma ^{2}_{G}}$, when present.

The ratio $\sigma ^{2}_{\textit {Imp}}/{\sigma ^{2}_{G}}$ defines the proportion of total genetic variance explained by imprinting. This ratio is, to some extent, equivalent to the definition of ${R^{2}_{e}}$ in [21], with the only difference being that these authors were interested in a broader concept of epigenetic mechanism while here we are interested in imprinting only. We graphically illustrate how imprinting can impact the evaluation of marked variance, and its consequences if ignored. We set *a*=2 and let the imprinting effect *i* vary between 0 (no imprinting) and *a* (complete imprinting) according to the previously described imprinting model. Four different values were assigned to the dominance effect *d*: 0, $\frac {1}{4}a$, $\frac {1}{2}a$ and *a*, representing from no dominance to complete dominance. Allele frequency *p* for the *A*_1_ allele varied from 0 to 1.

**Fig. 2:**
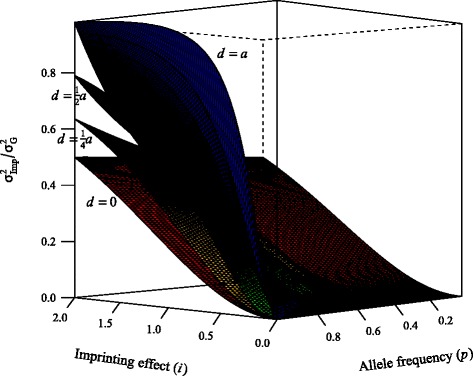
Proportion of genetic variance contributed by imprinting as a function of allele frequency (*p*), dominance level (*d*), and imprinting effect (*i*)

As shown in Fig. 2, ${R^{2}_{e}}=\sigma ^{2}_{\textit {Imp}}/{\sigma ^{2}_{G}}$ increases with *i*, *d* and *p*. When *d*=0, ${R^{2}_{e}}$ does not vary with *p* since in this case

$${R^{2}_{e}} = \frac{2pqi^{2}}{2pqa^{2}+2pqi^{2}}=\frac{i^{2}}{a^{2}+i^{2}}. $$

Under dominance, allele frequency drives ${R^{2}_{e}}$ from small values at lower allele frequency to large values at higher frequency, with more pronounced effects with larger values of *d*. When imprinting effects are small (e.g., $i < \frac {1}{4}a$), it seldom accounts for more than 10 % of the genetic variance, unless *p* is close to 1 and *d* is close to *a*.

**Fig. 3:**
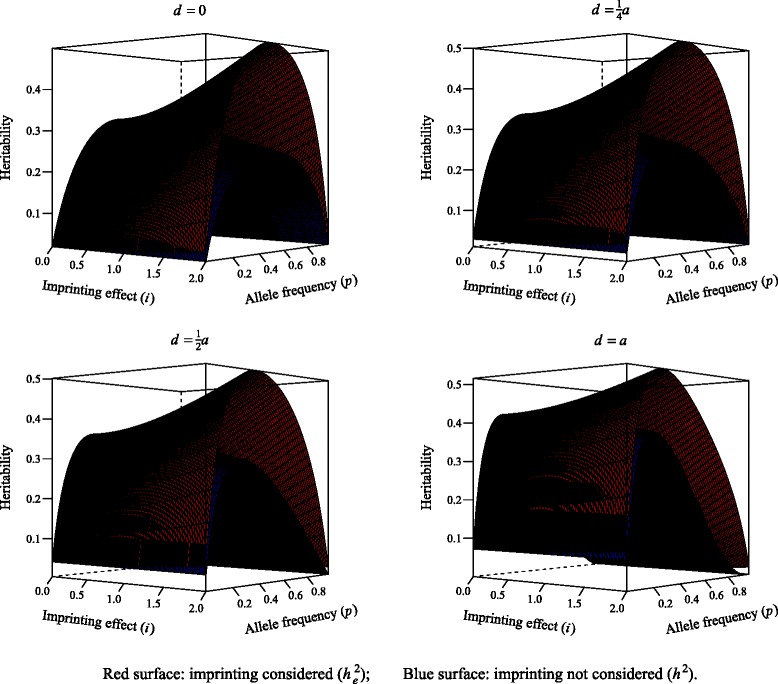
Narrow sense heritability with consideration of imprinting (${h^{2}_{e}}$, red surface) or without it (*h* ^2^, blue surface) as a function of allele frequency (*p*) and imprinting effect (*i*) at various level of dominance

Figure 3 shows how narrow sense heritability changes with (imprinting model) or without (additive model) consideration of imprinting at the four values of *d*. The environmental variance was set to ${\sigma ^{2}_{e}}=4$ across all situations and it was assumed that there was no interaction between environmental and genetic factors; *a*, *i*, *d* and *p* were as before. The additive variance obtained with imprinting was always larger than when an additive model was employed, as expected by construction. If we denote “epigenetic heritability” as ${h^{2}_{e}}$ [21] and that without consideration of imprinting as *h*^2^, the difference between ${h^{2}_{e}}$ and *h*^2^ is maximum when imprinting is at its highest level. This is not surprising because the larger *i* is, the higher the proportion of additive variation (i.e., ${\sigma ^{2}_{A}}$) accounted for by imprinting is (Eq. 2). Thus, if imprinting is present, the standard additive model would capture only part of the additive variance, resulting in an underestimate of the potentially markable variation.

## Mouse data analysis – materials and methods

The preceding discussion on the quantitative imprinting model applies to a single locus only and does not guide on how imprinting would contribute to heritability of a complex trait, presumably affected by multiple loci and with many of these not imprinted at all. Also, it is unknown how imprinting affects estimates of heritability when it is ignored in the estimation procedure. Hence, a real data analysis was performed to evaluate the impact of imprinting on a quantitative trait.

Previous studies have suggested that obesity-related traits could be affected by imprinting in both humans [28, 29] and mice [30]. Hence, mouse body mass index (BMI, defined as body weight divided by the square of tailless body length), considered to be a good indicator of obesity status, was chosen as the target trait in this analysis. The mouse dataset (build 37), generated for a series of studies on obesity and diabetes, was downloaded from The Wellcome Trust Centre for Human Genetics website (http://mus.well.ox.ac.uk/mouse/HS/). This population was obtained by crossing eight inbred strains followed by 50 generations of approximately random mating. BMI measurements were pre-corrected for body weight, season, month and day for a total of 1,940 *F*_2_ individuals (168 full-sib families), with more than 12,000 genotyped SNP markers located on 19 autosomes. BMI values seemed normally distributed with mean −0.4568 (nagative values were due to pre-correction on original data) and variance 0.0357. Additional descriptions of the data are in the website and in [31].

To test the effect of imprinting, one must be able to distinguish two heterozygous genotypes, which is impossible if conventional coding systems used in GWAS or whole-genome prediction studies (e.g., genotypes *A*_2_*A*_2_, *A*_1_*A*_2_/*A*_2_*A*_1_ and *A*_1_*A*_1_ coded as −1, 0, and 1, respectively) are adopted, because *A*_1_*A*_2_ and *A*_2_*A*_1_ are not differentiated. To make *A*_1_*A*_2_ and *A*_2_*A*_1_ distinguishable, marker genotypes (in the form of *AA*, *AB*, *BB*) were fed into BEAGLE 3.3.2 [32, 33] for sporadic missing genotype imputation and haplotype phase inference. This software can perform haplotype inference of unphased (unknown parental origin) genotypic data using linkage information between marker genotypes, with or without pedigree information, and give an inferred phased (known parental origin status) genotype as an output. With phased genotype, markers can be coded as described below. After filtering markers with minor allele frequency (MAF) less than 0.05, 10,021 markers were kept for analysis.

One objective of this study is to assess the consequences of “erroneously” using an additive model without considering imprinting in GWAS if imprinting does affect that trait. Therefore, the data was analyzed using regression models with or without imprinting, as described below. First, according to the imprinting model (Fig. 1), the following matrix can be used to associate different genetic effects with the four possible genotypes [3, 34, 35]:

(3) $$ \mathbf{S} = \begin{array}{cc} \overset{{\scriptsize{\mathbf{I}_{a}}}\;\;\;\, {\scriptsize{\mathbf{I}_{d}}} \;\;\;\, {\scriptsize{\mathbf{I}_{i}}}}{\left(\begin{array}{ccc} 1 & 0 & 0 \\ 0 & 1 & 1 \\ 0 & 1 & -1 \\ -1 & 0 & 0 \\ \end{array}\right)}& \begin{array}{ll} \scriptsize{\longleftarrow} & \scriptsize{A_{1}A_{1}}\\ \scriptsize{\longleftarrow} & \scriptsize{A_{2}A_{1}}\\ \scriptsize{\longleftarrow} & \scriptsize{A_{1}A_{2}}\\ \scriptsize{\longleftarrow} & \scriptsize{A_{2}A_{2}}\\ \end{array} \end{array},   $$

where **I**_*a*_, **I**_*d*_, and **I**_*i*_ are vector indicators for the additive (*a*), dominance (*d*), and imprinting (*i*) effects in the four genotypes, respectively. Using this coding matrix, models with an additive effect only, additive and dominance, and additive plus dominance plus imprinting can be written in matrix form as:

(4) $$\begin{array}{*{20}l} \mathbf{y} &= \mathbf{1}\mu + \mathbf{Xb} + \mathbf{I}_{a}\beta_{1} \phantom{{}+ \mathbf{I}_{d}\beta_{2} + \mathbf{I}_{i}\beta_{3}} + \mathbf{Zu} + \mathbf{Qc} + \mathbf{e}, \end{array} $$

(5) $$\begin{array}{*{20}l} \mathbf{y} &= \mathbf{1}\mu + \mathbf{Xb} + \mathbf{I}_{a}\beta_{1} + \mathbf{I}_{d}\beta_{2} \phantom{{}+ \mathbf{I}_{i}\beta_{3}} + \mathbf{Zu} + \mathbf{Qc} + \mathbf{e}, \end{array} $$

(6) $$\begin{array}{*{20}l} \mathbf{y} &= \mathbf{1}\mu + \mathbf{Xb} + \mathbf{I}_{a}\beta_{1} + \mathbf{I}_{d}\beta_{2} + \mathbf{I}_{i}\beta_{3} + \mathbf{Zu} + \mathbf{Qc} + \mathbf{e}, \end{array} $$

where **y** is an *n*-element vector containing the observations; *μ* is the population mean common to all individuals; **X** is the incidence matrix relating the vector **y** to the vector of fixed effects **b** (sex, litter size and cage density); *β*_1_, *β*_2_ and *β*_3_ are regression coefficients that are interpreted as additive, dominance, and imprinting effects, respectively; **u** is the vector of infinitesimal additive effect with associated incidence matrix **Z**, and it is assumed that $\mathbf {u}\sim N\left (\mathbf {0}, \mathbf {A}{\sigma ^{2}_{u}}\right)$, where **A** is the additive relationship matrix calculated from the pedigree and ${\sigma ^{2}_{u}}$ is the infinitesimal additive genetic variance; **c**, with associated incidence matrix **Q**, is the vector of normally and independently distributed random effects represented by different cages in which an individual is raised, and it is assumed that **c** has a zero mean and homogeneous variance ${\sigma ^{2}_{c}}$; **e** is the vector of model residual, whose elements are assumed to be normally and independently distributed with zero mean and homogeneous variance ${\sigma ^{2}_{e}}$.

**Fig. 4:**
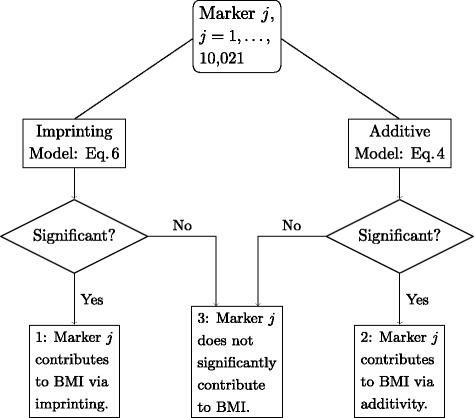
Workflow for data analysis

A likelihood ratio test (LRT) between Models 6 and 5 tests the significance of *β*_3_, which represents the imprinting effect *i*; a LRT between Models 5 and 4 tests the significance of *β*_2_, the dominance effect *d*; and a LRT between Model 4 and a null model without marker information tests the significance of *β*_1_, interpreted here as the allelic substitution effect *α*. This procedure of data analysis is graphically represented in Fig. 4. The main objective of this study was to compare a model with imprinting with the common GWAS strategy used today (i.e., considering additive but not dominance effect) to evaluate the extent to which imprinting affects inference on marked variance. The marked variance ignoring imprinting was assessed as

(7) $$ \hat\sigma^{2}_{\text{SNP}} = \sum_{j\in\mathrm{Box\:2}}\hat\sigma^{2}_{Men,j} = \sum_{j\in\mathrm{Box\:2}}2\hat p_{j}\hat q_{j}{\hat\alpha^{2}_{j}},   $$

using only markers falling in Box 2 of Fig. 4, where $\hat p_{j}$ and $\hat q_{j} = 1-\hat p_{j}$ are maximum likelihood estimates of allelic frequency at marker locus *j*. With consideration of imprinting, the marked variance would be

(8) $$ \begin{aligned} \hat\sigma^{2}_{\text{SNP}} &= \sum_{j\in\mathrm{Box\:2}}\hat\sigma^{2}_{Men,j} + \sum_{j\in\mathrm{Box\:1}}\left(\hat\sigma^{2}_{Men,j} + \hat\sigma^{2}_{Imp,j}\right) \\ &= \sum_{j\in\mathrm{Box\,2}}2\hat p_{j}\hat q_{j}{\hat\alpha_{j}^{2}} + \sum_{j\in\mathrm{Box\,1}}2\hat p_{j}\hat q_{j}\left({\hat\alpha_{j}^{2}}+\hat {i}_{j}^{2}\right), \end{aligned}   $$

using “imprinted markers” (Box 1) and “unimprinted markers” (Box 2). In both cases, linkage equilibrium between markers was assumed.

In order to deal with potential problems raised by multiple testing in single marker regression, the *p*-value for individual testing was set to 1.316×10^−5^ to obtain a 0.05 genome-wide type I error rate using the Šidák’s correction. The effective number of independent tests used in the multiple testing correction was calculated using LD information between markers based on the method described in [36]. LD (measured by *r*, the pairwise haplotypic Pearson’s correlation coefficient) between marker pairs across the whole genome was calculated using the R package genetics [37]. Models were fitted using R package pedigreemm [38, 39] with variance components of random effects estimated via restricted maximum likelihood (REML).

## Results and discussion

### Significant markers and marked variance

After data cleaning, 10,021 SNP markers were kept for the whole genome scan using methods described in the previous section. As a result, 7 markers were additively significant, and 11 markers were significant for an imprinting effect, either from the paternal side or from the maternal side. The latter suggests the markers are linked with imprinted genes or QTLs. Therefore, all discussions of “imprinted markers” hereafter should be interpreted accordingly. Because many adjacent markers showed co-significance due to high LD (*r*^2^ between markers > 0.99), information redundancy exists for these markers. In order to assess the variance explained by each genomic region, we chose only one marker from each highly correlated marker cluster. After this filtering, only 3 markers were additively significant and 6 were significant for imprinting, 4 of which were paternally imprinted and 2 were maternally imprinted (Table 1). Imprinting direction (i.e., maternal imprinting or paternal imprinting) was determined from the signs of $\hat \alpha $ and $\hat i$. This is because, according to the imprinting model and the genotype codes, the maternal and paternal allelic substitution effects are written as *α*−*i* and *α*+*i*. Since reduced expression induced by imprinting indicates a smaller absolute value of the parental substitution effect, a maternal imprinting is then suggested if $\hat \alpha $ and $\hat i$ have the same sign, and a paternal imprinting is suggested if these two estimates have different signs. This depends on how the four genotypes are coded and one may obtain a reversed result if imprinting is coded oppositely. With these “uniquely” significant markers, marked variance was then computed according to Eqs. 7 and 8. We found that $\hat \sigma ^{2}_{\text {SNP}}$ with and without consideration of imprinting was 1.218×10^−4^+0.624×10^−4^=1.842×10^−4^ and 1.218×10^−4^, respectively. The variances explained by the infinitesimal and random cage effects were 3.816×10^−4^ and 4.742×10^−4^ for the imprinting model, and 3.805×10^−4^ and 4.747×10^−4^ for the additive model, respectively. Residual variance was about 1.869×10^−3^ for both cases. Since there were estimates of variance components (i.e., ${\hat \sigma ^{2}_{u}}$, ${\hat \sigma ^{2}_{c}}$ and ${\hat \sigma ^{2}_{e}}$) for every marker, the estimates reported here were the average over 10,021 estimates. Small standard deviations of REML estimates in both models indicated that estimates were fairly precise. Variance components estimates are presented in Table 2 along with their asymptotic standard deviations. Values in Table 2 indicated that marked variance was increased by 50 % in this GWAS-like whole genome scan if variation due to imprinting was considered. In other words, if existing imprinting was not accounted for, about one third marked variation would be lost, potentially leading to wrong conclusions in genetic analysis using SNP markers.

**Table 1:** Significant markers and imprinting status when imprinting was accounted for

Marker	Chr.	Closest QTL ^*a*^	Status ^*b*^	*p*-value ^*c*^	$\hat \alpha \;(\times 10^{-3})$	$\hat i\;(\times 10^{-3})$
rs3697020	2	*T2dm3* (within)	A	1.476 ×10^−6^	11.63±2.41	-
rs3676388	2	*T2dm2sa* (within)	A	9.357 ×10^−6^	−9.99±2.26	-
rs3726626	15	*W3q6* (within)	A	2.987 ×10^−6^	9.30±1.97	-
rs3662117	2	*Gnf1* (within)	M	4.061 ×10^−6^	2.51±2.61	6.20±2.10
rs6212614	3	*Orgwq5* (within)	M	6.197 ×10^−6^	2.11±1.96	4.18±1.59
rs13476734	2	*T2dm3* (within)	P	1.302 ×10^−5^	−1.36±2.05	4.59±1.79
rs6371982	3	*W10q3* (within)	P	5.055 ×10^−6^	1.05±1.94	−4.36±1.59
rs3665109	3	*W10q3* (within)	P	8.690 ×10^−6^	−1.82±1.92	4.00±1.62
gnf04.110.360	4	*W10q10* (within)	P	9.365 ×10^−6^	1.79±1.96	−4.17±1.62

^a^Information from the Mouse Genome Informatics website (http://www.informatics.jax.org/) ^b^A: additively significant; M: maternal imprinting; P: paternal imprinting ^c^ *p*-value threshold was set to 1.316×10^−5^ to ensure a 0.05 whole-genome type I error rate with Šidák’s correction

**Table 2:** Variance components estimates (×10^−4^) using models with (Imp) or without (Add) imprinting effect

Model	$\hat \sigma ^{2}_{\text {SNP}}$	${\hat \sigma ^{2}_{u}}$ (infinitesimal)	${\hat \sigma ^{2}_{c}}$ (cage)	${\hat \sigma ^{2}_{e}}$ (residual)
Imp	1.842	3.816±0.762	4.742±0.344	18.687±1.36
Add	1.218	3.805±0.793	4.747±0.360	18.694±1.37

### Interpretation of detected markers

In this study, we found 3 markers that are additively related to mouse BMI, all of which are related to a certain QTL that has an effect on mouse body weight or diabetes. Particularly, marker rs3697020 is located in a diabetes related QTL *T2dm3* (type 2 diabetes mellitus 3, chromosome 2) that is also highly interactive with obesity [40]; marker rs3676388 is located in another diabetes related QTL *T2dm2sa* (type 2 diabetes mellitus 2 in SMXA RI mice) on the same chromosome [41]; lastly, marker rs3726626 is located in a body weight related QTL *W3q6* (weight 3 weeks QTL 6) on chromosome 15 [42]. Although the main effect of QTL *T2dm3* and *T2dm2sa* is related to the development of type II diabetes in mice, both are highly correlated with obesity status in mice [40, 41], which is commonly considered as a high risk of developing diabetes. Since the data used here was generated for a series of studies on mice diabetes, it was not surprising that markers associated with diabetes-related QTL were detected.

All 6 presumably imprinted markers detected in our analysis are located in the vicinity of QTLs associated with body weight or growth. For example, marker rs3662117 is in *Gnf1* (growth and fatness 1), a QTL located on chromosome 2 that has a large impact on growth and body composition [43]. Marker rs6212614 resides in *Orgwq5* (organ weight QTL 5, chromosome 3), a QTL affecting organ weight in mouse [44]. This pleiotropic QTL has an impact on limb bone length as well, such that it may potentially affect body length and hence influence body mass index. Markers rs6371982, rs3665109, and gnf04.110.360 are located in *W10q3* (weight 10 weeks QTL 3) and *W10q10* (weight 10 weeks QTL 10) on chromosomes 3 and 4 respectively, which are two QTLs affecting mouse body weight at the age of 10 weeks [42]. Interestingly, markers rs3697020 and rs13476734 are both in QTL *T2dm3*, but one is additively significant and the other has a strong imprinting effect. Since the distance between these two markers is large (about 5 Mb), it is possible that these two markers are capturing different signals (see below). Same as the additive markers, locations of these presumably imprinted markers indicated that variation on BMI is likely an inherited feature of variation on body weight and body length via the major QTLs.

We also checked whether these 6 presumably imprinted markers are located in any known imprinted regions. It was found that 5 out of 6 are in the genomic region of reported imprinted genes or *i*QTLs (imprinted QTL). Specifically, markers rs6371982 (chromosome 3, 16.96 cM) and rs3665109 (chromosome 3, 19.81 cM) are both in the range of *i*QTL *Wti3.1* (chromosome 3, 3.79 ∼32.75 cM), which has a significant effect on most mouse body weights from week 1 to 9 and is expressed from the maternally inherited allele [3]. Marker rs6212614 (chromosome 3, 60.92 cM) is located in the range of another weight related *i*QTL *Wti3.2* (chromoeome 3, 60.71 cM), which was also reported in [3]. Marker rs13476734 (chromosome 2, 60.01 cM) is adjacent to a maternally expressed imprinted gene *Gatm* (glycine amidinotransferase, 60.63 cM) [45]. This gene encodes a metabolic enzyme involved in creatine synthesis, which plays an important role in embryonic and fetal growth as well as brain functioning [46]. Marker rs3662117 (chromosome 2, 75.95 cM) is adjacent to a paternally expressed protein coding gene *Mcts2* (malignant T cell amplified sequence 2, 75.41 cM) [45], which influences the choice of polyadenylation (poly A) site for transcripts of the host gene *H13* in an allele-specific manner [47]. However, no strong evidence regarding the effect of *Mcts2* on body weight, obesity, or diabetes has been reported. Besides these five markers, marker gnf04.110.360 (chromosome 4, 56.49 cM) does not fall in any known imprinted region. However, it is located in a genomic region that is predicted to harbor three maternally expressed genes [48]. These genes are *4931406I20Rik* (53.44 cM), *Krc* (55.51 cM), and *Grik3* (58.91 cM). There are also two genes adjacent to this interval that are predicted to be paternally expressed (*Ftl2* and *AU040320*), but the positions of these two genes are outside of the maternally expressed region (58.94 cM and 60.94 cM, respectively). Therefore, these two intervals are likely two adjacent clusters that have different imprinting directions, and the imprinting direction of this marker detected in our study matched with previous findings. Unfortunately, no evidence indicating association between body weight and these three genes has been reported.

Our analysis indicated that in this particular data set, markers associated with mouse BMI through either additivity or imprinting can be effectively detected, and the functions of the genes or QTLs harboring these markers supported our discovery on the marker-trait association. Elevated estimates of marked variances suggested that, by incorporating imprinting effects in to a quantitative genetic model, the proportion of phenotypic variance explained by significant markers increased noticeably. In addition to three markers detected using the additive model, six markers were deemed associated with an imprinting effect when the phenomenon was accounted for; the directions of the imprinting effects of all six markers were consistent with previously reported studies. This indicated that the imprinting model detected extra variation that the additive model was not able to capture, so a higher estimate of marked variance was obtained. However, this result was achieved by adding variances contributed by markers from distinct single marker regression models, which may give a misleading picture of the variance captured by markers because LD between them may overemphasize the contribution of significant markers [49]. Although only one marker in each high LD cluster was kept for calculating marked variance in order to reduce bias, caution still needs to be exercised when interpreting this variance since it was obtained from unshrunken estimates of marker effects with simple regression approaches.

### Validation of imprinting detection – a simulation

Besides a potential inflation of marker-explained variance stemming from LD between markers, it should also be noted that the detection of imprinting relies mainly on the comparison between heterozygotes, which might be confounded by dominance under some circumstances. For example, even though columns **I**_*d*_ and **I**_*i*_ in the **S** matrix (Eq. 3) are ideally orthogonal, there might be a large collinearity if heterozygotes are mostly say, *A*_1_*A*_2_, and hence hampering estimability of either the dominance or the imprinting effect. If, on the other hand, the two heterozygote types have similar frequencies in the population, both dominance and imprinting effects are identifiable and the estimates of the two effects would be uncorrelated, ideally. Thus, the results presented in the previous section would be more convincing if the detection of imprinting was not affected by dominance.

In order to test for potential confounding between imprinting and dominance, we performed the following simulation. First, a population of 5,000 unrelated individuals was created. For each individual, we generated 500 biallelic loci in linkage equilibrium, with allele frequencies varying over {0.05,0.10,0.15,…,0.90,0.95}. One hundred out of the 500 loci were randomly selected to have additive effects generated from a standard normal distribution. Within these 100 loci, 50 and 10 were randomly chosen to have dominance and imprinting effects, respectively, both generated from a standard normal distribution. Note that some loci may have all three true effects since we did not force the two sets of loci with either dominance or imprinting effects to be mutually exclusive. Genotypic values at each of the loci that had an effect were created according to Fig. 1, given the genotype at that locus. Environmental effects were drawn from a normal distribution with zero mean and variance equal to the variance among genotypic values so the heritability was roughly 0.5.

We fitted Models 5 and 6 to the simulated data, as well as the following model where the dominance effect was not accounted for

(9) $$ \mathbf{y} = \mathbf{1}\mu + \mathbf{Xb} + \mathbf{I}_{a}\beta_{1} + \mathbf{I}_{i}\beta_{3} + \mathbf{Zu} + \mathbf{Qc} + \mathbf{e}.   $$

**Fig. 5:**
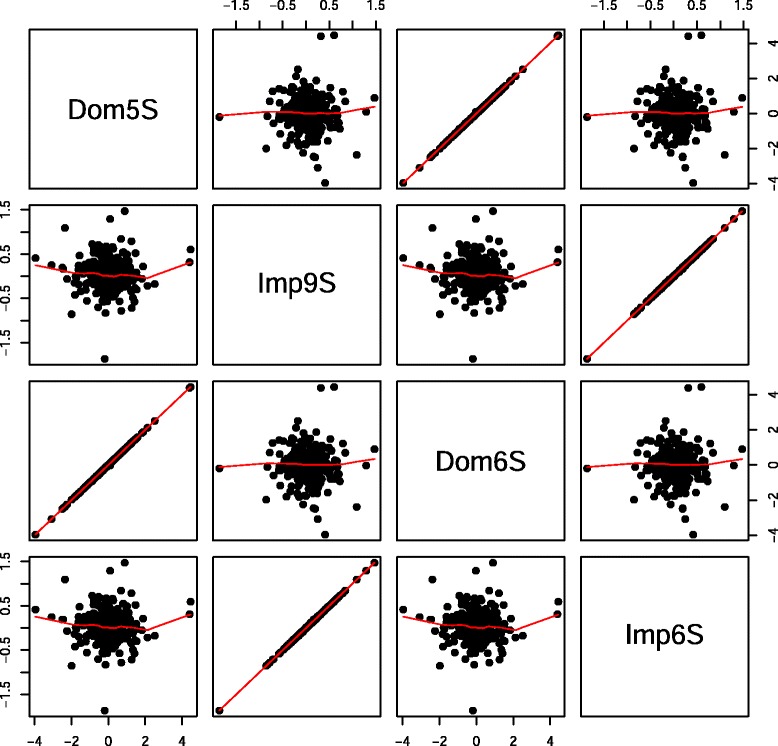
Paired scatter plot of estimated dominance and imprinting effects using simulated imprinting data. Dom5S and Dom6S are estimated dominance effects using Models 5 and 6, respectively; Imp9S and Imp6S are estimated imprinting effects using Models 9 and 6, respectively

**Fig. 6:**
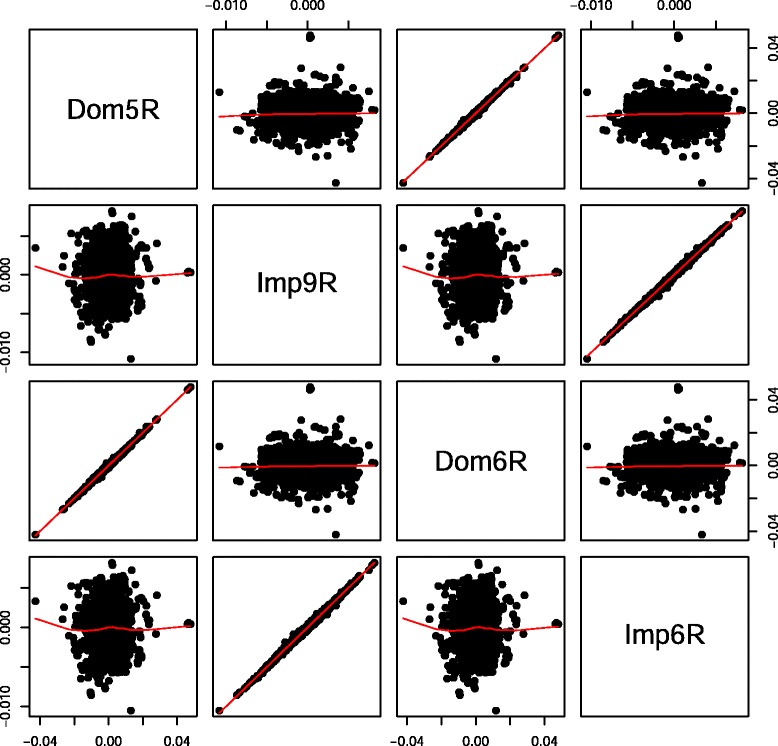
Paired scatter plot of estimated dominance and imprinting effects using real mouse data. Dom5R and Dom6R are estimated dominance effects using Models 5 and 6, respectively; Imp9R and Imp6R are estimated imprinting effects using Models 9 and 6, respectively

The reason for fitting Model 9 is that, because **I**_*d*_ and **I**_*i*_ are orthogonal, we expect the estimate of *β*_3_ (representing *i*) from this model should be equal to that obtained from Model 6, conditional on the additive effect *β*_1_. We then compared the estimates of imprinting effects from Models 6 and 9 and dominance effects from Models 6 and 5. As shown in Fig. 5, regardless of whether estimated separately or jointly, the dominance and imprinting effects were uncorrelated to each other, reflecting that the population is under Hardy-Weinberg equilibrium. When using the real mouse data, the same picture emerged (Fig. 6). Therefore, inferences on the imprinting effect in this current data set are unlikely to be confounded by dominance.

**Fig. 7:**
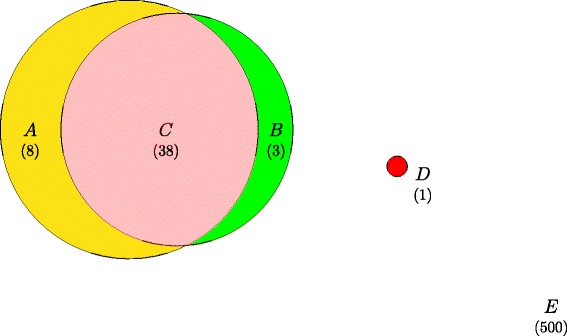
Venn’s diagram illustrating simulation results. The total number of loci was 500 (*E*) and 100 had a true effect. 46 loci were detected as additive (*A* plus *C*) using the Dom data, and 41 were found (*B* plus *C*) using the Imp data. The same model testing procedure found 38 additive loci in common (*C*) using two data sets, indicating that imprinting does not have a big impact on detecting additive loci. One locus was found to be imprinted (*D*) using the Imp data and none were detected using the Dom data

Apart from a potential confounding between imprinting and dominance, we were also interested in testing whether the LRT that was applied to test for significant imprinting effect would pick up any unexisting imprinting effect as a false discovery. To do this, we took the same simulated population as described above but generated the genotypic values by including only simulated additive and dominance effects (i.e., without adding the simulated imprinting effect). We denote this data as the Dom data and referred to the one with true imprinting effects as the Imp data. Then we fitted Models 4 and 9 to the Dom data to evaluate how imprinting could be detected in a population not affected by imprinting and compared to the result obtained when the same procedure was applied on the Imp data. As a result, one locus showing significant imprinting effect was detected in the Imp data (*p*-value 3.49×10^−16^) and none were detected using the Dom data, as expected. As a comparison, the smallest *p*-value obtained when testing for imprinting using the Dom data was 0.0043, ranked only in the 9^th^ place if imprinting was tested using the Imp data. Considering that there were only 10 loci with a true imprinting effect in the simulation, a locus with *p*-value ranked in the 9^th^ place would not be detected if the significance threshold was set appropriately. Therefore, it seemed unlikely that a locus would be spuriously claimed as “imprinted” if the true imprinting was absent. Also, the existence of imprinting did not have a large impact on detecting an additive effect, since the detected additive loci using either Dom or Imp data were largely overlapping (Fig. 7).

Through simulation, we corroborated that, in general, imprinting would not be erroneously claimed if it does not exist and would not be confounded by dominance in a population under Hardy-Weinberg equilibrium. Therefore, it is likely that the higher estimate of marker-explained variance in the mouse population was indeed due to imprinting. Hence, the failure of capturing variation attributed to existing imprinting using an additive model may lead to an underestimate of marked variance.

### Elevated marked variance – just because of more markers?

We found that incorporating genomic imprinting in GWAS produced a larger estimate of phenotypic variance accounted for by significant markers. However, when we estimated the marked variance under imprinting, additively significant markers were also included. Therefore, one may argue that, by construction, the marked variance under imprinting would always be larger as long as markers significant on imprinting are detected (Eq. 7 versus 8). Because an objective was to assess how imprinting affects the estimate of marked variance, the following evaluation was considered: we took all markers listed in Table 1, but instead of using a “correct” model, we used a “wrong” model to re-estimate the variance accounted for by these markers, i.e., if one marker was detected as imprinted, we now use an additive model to estimate the marked variance, and vice versa for the additively significant markers. The results of this procedure are in Table 3.

**Table 3:** Marked variance when marker effects were estimated using the correct (italic) and the wrong models

Detected	Model used to estimate	Marked variance (×10^−4^)
marker (No.)	marker effects	
Additive (3)	*Additive*	*1.218*
	Imprinting	1.231
Imprinted (6)	Additive	0.091
	*Imprinting*	*0.624*

When markers are imprinted, as indicated in Table 1, “erroneously” using an additive model produced a much lower estimate of marked variance (decreased by 85 %). This is because the variance of a locus under the imprinting model is 2*pq*(*a*+(*q*−*p*)*d*)^2^+2*pqi*^2^ (Eq. 2), where the term 2*pqi*^2^ (≠0 if imprinting exists) is due to imprinting. If an additive model was used, this term would be lost, producing a lower estimate of marked variance. If a marker shows additivity but not imprinting, on the other hand, it would be expected that *i*=0 and hence applying an imprinting model on an additive marker would not increase the variance. However, in practice, it is unlikely that the estimate of *i* is exactly zero, and hence using a “wrong” model on an additive maker may give a slightly higher variance. However, the difference is negligible (1.218×10^−4^ versus 1.231×10^−4^, Table 3).

### Can imprinting explain missing heritability?

As stated before, heritability is an important parameter in genetic analysis and is usually taken as $2pq\alpha ^{2}/{\sigma ^{2}_{P}}$ at a single locus. In GWAS, summation of 2*pqα*^2^ across all significant markers gives the total marked additive genetic variance under the assumption of linkage equilibrium between markers. The ratio between this total marked variance and phenotypic variance ${\sigma ^{2}_{P}}$ is usually referred to as the “bottom up” heritability in GWAS [50, 51]. However, marked additive genetic variance differs from additive genetic variance (e.g., [52, 53]). Therefore, one needs to be cautious when interpreting this “marked” variance, and it is often observed in GWAS that heritability estimated using only statistically significant markers is much lower than pedigree derived heritability, termed as “top-down” estimate in some literature (e.g., [50]). This issue is commonly known as the “missing heritability” problem [54]. For example, human height is a trait with estimates of heritability from family studies as high as 0.8 [55, 56], but the variation captured by significant SNP markers from GWAS may take only a proportion of 5∼10 *%* of the total [57–59].

Finding sources of missing heritability has been a topic of much interest in genetic and epidemiological studies. The most obvious and likely explanation for this phenomenon is that most traits are polygenic and that markers are in incomplete LD with QTLs as illustrated in [60]: ${h^{2}_{M}}$, the proportion of marker-explained variation, is always smaller than *h*^2^, unless the SNP markers can explain all genetic variation due to perfect LD between markers and causal loci (or in rare cases where some SNP markers are the causal loci themselves), in which case ${h^{2}_{M}}=h^{2}$. Unfortunately, this situation is unlikely to be encountered in practice. Further, if only genome-wide-significant (GWS) markers are used in genetic analysis, the variation captured by the significant markers ($h^{2}_{\textit {GWS}}$) would be even smaller, resulting in a large amount of missing heritability, measured by $h^{2}-h^{2}_{\textit {GWS}}$ [50, 60]. Therefore, a more appropriate approach could be combining information from both significant markers and pedigree that reflects a “major gene model” situation commonly observed in complex traits analysis, where markers represent the major gene part and pedigree represents the infinitesimal part. Further, although much missing heritability can be recovered by simultaneously including all available dense markers in a statistical model [61], the upper bound of this improvement is ${h^{2}_{M}}$, indicating that the variation hidden by incomplete LD relationships between markers and QTLs is “still missing” and difficult to be restored [60]. The covariance between alleles stemming from LD complicates the variance assessment, and epistatic effects, i.e., interactions between causal loci are often ignored. These two issues can also lead to dubious attributions of genetic variation [49, 62].

Epigenetic variation has been suggested as another potential source of missing heritability (e.g., [63, 64]). From the imprinting model introduced above, it is expected that imprinting may have an impact on additive genetic variance of a single locus, and hence affect the bottom up estimate of heritability, as evidenced in our analysis. Combining the bottom-up and top-down genetic variation may lead to a less clear result since infinitesimal effects contributed more to the additive genetic variance than markers, and the estimates of this component were close to each other when using two approaches (Table 2). Nevertheless, incorporating imprinting still resulted in a 10 % increase on heritability, as the estimates with and without consideration of imprinting using variance components in Table 2 are

$$\hat{h}^{2}_{\text{Imp}} = \frac{1.842+3.816}{1.842+3.816+4.742+18.687}=0.195, $$

and

$$\hat{h}^{2}_{\text{Men}} = \frac{1.218+3.805}{1.218+3.805+4.747+18.694}=0.176, $$

respectively. Thus, a higher estimate of additive variability was found whether the pedigree information was included or not. This result indicated that the underestimate of additive variation by erroneously using an additive model in the case of imprinting could be a potential source of missing heritability in GWAS, as discussed in [63, 64].

### Imprinting effect and parent-of-origin effect

Our results indicated that existing imprinting effects should not be ignored in genetic analysis. Meanwhile, it is also important to make a distinction between the terms “imprinting effect” and “parent-of-origin effect”. These terms are often used exchangeably in much of the epigenetic literature. However, a parent-of-origin effect referring to different genetic contribution of different parents to offspring is a broader concept than an imprinting effect. Genomic imprinting is the most important source of parent-of-origin effects, but not the only one. For example, maternal effects observed in swine production is a well known form of parent-of-origin effect that is not known to involve any epigenetic mechanisms; reciprocal effects observed in poultry breeding is another type of parent-of-origin effect. In the imprinting model that was adopted in our analysis, all inferences were performed at the DNA level using SNP markers. Hence, not all “putatively” imprinted loci were necessarily caused by imprinting at the epigenetic level. Moreover, other factors can lead to the detection of spurious imprinting effect that is actually caused by other types of parent-of-origin effect (e.g., [65–67]) or even by linkage disequilibrium between markers [68]. Therefore, results from this study should be viewed as parent-of-origin effects instead of imprinting. On the other hand, if the objective of a certain study is to determine or verify imprinting status, we recommend that examination of variation must be taken place at the epigenetic level using, for instance, differential methylation analysis. However, this does not contradict the statement that an underestimate of additive variability would occur if existing imprinting was ignored.

## Conclusion

We were inspired by studies that proposed equivalent one-locus imprinting models for quantitative genetic analysis. These studies defined paternal and maternal gene substitution effects. As such, imprinting does contribute to additive variance and a partition of additive variance into unimprinted and imprinted components is available. This variance decomposition hints that heritable genetic variation induced by epigenetic mechanisms, especially genomic imprinting, may have a considerable impact on the underlying genetic architecture of some complex traits, but it is largely neglected in many studies. Specifically, narrow sense heritability, especially marked “bottom up” heritability in GWAS, may be underestimated if one ignores imprinting when it is present. We tested this using a genome-wide association study performed on mouse BMI data. Results indicated that the portion of phenotypic variation explained by significant SNP markers increased drastically when imprinting effects were considered.

Moreover, the imprinting regression model used here detects differences between paternally and maternally inherited alleles regardless of whether the biological mechanism is imprinting or not. Hence, this model might be capturing other (either genetic or epigenetic) mechanisms that produce nonequivalent contributions of paternal and maternal genomes as well. Therefore, it would be more appropriate to refer to this model as a model incorporating parent-of-origin effects, and such that, it can be applied to a number of situations. For example, in the human genome, more than 50 % of the genes have shown preferential expression of the paternal or maternal allele due to various mechanisms [69], indicating that our approach may apply to a wide range of complex traits, whenever reciprocal heterozygotes generate different genotypic values. Since imprinting is only one of such mechanisms, it is possible that more (epigenetic) sources of phenotypic variation and of missing heritability may be uncovered in the future. Nevertheless, imprinting is widely considered as the most important source of parent-of-origin effects, so in order to avoid a possibly wrong inference on genetic architecture of a complex trait of interest, imprinting should not be neglected if indication of the presence of imprinting exists.

## Abbreviations

AS: Angelman syndrome
BMI: Body mass index
cM: Centi Morgan
GWAS: Genome‐wide association studies
GWS: Genome‐wide-significant
LD: Linkage disequilibrium
LRT: Likelihood ratio test
MAF: Minor allele frequency
PWS: Prader‐Willi syndrome
QTL: Quantitative trait loci
REML: Restricted maximum likelihood
SNP: Single nucleotide polymorphism
